# Validating adverse events in administrative healthcare data in Ireland: a retrospective chart review study

**DOI:** 10.1186/s12913-025-13201-x

**Published:** 2025-08-20

**Authors:** Anna Connolly, Maria Unbeck, Fiachra Bane, Kasia Bail, Margaret Craig, Anne Matthews, Anthony Staines, Marcia Kirwan

**Affiliations:** 1 School of Nursing, Psychotherapy and Community Health, Dublin City University, Dublin, Ireland; 2 School of Health and Welfare, Dalarna University, Falun, Sweden; 3 Department of Clinical Sciences, Danderyd Hospital, Karolinska Institutet, Stockholm, Sweden; 4 Healthcare Pricing Office, Health Service Executive, Dublin, Ireland; 5 Centre for Ageing Research and Translation, University of Canberra, and Synergy Nursing and Midwifery Research Centre ACT Health Directorate, University of Canberra, Canberra, Australia

**Keywords:** Health administrative data, Adverse event, Risk management, Chart review, Nursing-sensitive patient outcomes, Patient safety, Validation study, Nursing

## Abstract

**Background:**

Pneumonia, urinary tract infections, pressure ulcers and delirium are adverse events that affect older inpatients. Accurate administrative data are key to improving patient safety and healthcare quality. The aim of the study was to validate Hospital In-Patient Enquiry (HIPE) data on the occurrence of pneumonia, urinary tract infections, pressure ulcers and delirium in older patients discharged from an acute hospital in Ireland through retrospective chart review.

**Methods:**

A cohort of one thousand randomly selected admissions of inpatients aged over 65 from a university, tertiary hospital in 2022 were reviewed using a two-stage retrospective chart review. The researchers, healthcare professionals and patient representatives co-produced a study-specific chart review protocol and data collection instrument. HIPE data were validated by comparing the chart review data to the HIPE data. Since HIPE only codes the presence of the respective adverse event once, the comparisons between the HIPE data and the chart review data were carried out at admission level.

**Results:**

Of the 1,000 admissions reviewed, 231 (23.1%) contained at least one adverse event. At event level, 373 adverse events were identified including 133 pressure ulcers in 71 admissions, 101 delirium episodes in 100 admissions, 84 pneumonia episodes in 79 admissions and 55 urinary tract infections in 52 admissions. Of the 302 adverse events found in chart review on admission level, 96 (31.8%) of these were coded in the HIPE data and flagged by the Hospital Acquired Diagnosis indicator. Compared with chart review data, the overall sensitivity of the administrative data was low, and the specificity was high. The positive predictive values varied, and the negative predictive values were generally high. In HIPE data, 42 adverse events were found that were not identified in the chart review.

**Conclusions:**

The results demonstrate that HIPE data may not accurately represent these specific adverse events as experienced by older patients. Improving the accuracy of these data may facilitate benchmarking of adverse events across hospitals and countries and provide opportunities for improvements in patient safety.

**Supplementary Information:**

The online version contains supplementary material available at 10.1186/s12913-025-13201-x.

## Introduction

Adverse events (AEs) present ongoing challenges to patient safety and healthcare quality. These events can be defined as incidents that result in harm to patients [1]. AEs occur in up to 68.5% of admissions [2] and are deemed preventable to a high extent [3]. AEs can result from acts of commission, acts of omission or delayed care [4]. Although some AEs may not be preventable [5], enhanced policy and practice may decrease AE occurrence and reduce healthcare costs with prevention costs generally much lower than the cost of harm [6].

Nursing-sensitive AEs are patient outcomes that are affected by processes or structures of nursing care, but for which nursing is not exclusively responsible [5]. Needleman et al. [7] identified 14 common nursing-sensitive patient outcomes including urinary tract infections (UTIs), pneumonia, and pressure ulcers. Along with delirium, these four outcomes are measurable indicators of the ‘Failure to Maintain’ (F2M) conceptual framework used to indicate the rationing of care that contributes to functional and cognitive decline in older patients during hospitalisation [8]. These AEs are commonly experienced by older patients [9, 10]. However, their visibility, and the visibility of nursing practice, are often limited in administrative data due to coding standards which can rely heavily on medical documentation and less so on nursing documentation [11]. The high incidence of these complications warrants attention, as just these four complications were associated with nearly a quarter of above average length of stay for older patients [12], and cost more than other kinds of patient complexity [13].

Understanding the true rates of these AEs is key to improving the visibility of nursing care and nursing-sensitive patient outcomes in administrative data, and to better understand potential cost savings for patient safety in hospital decision making.

AEs are disproportionately experienced by older patients in comparison to younger patients. Patients aged over 65 require more complex care due to co-morbidities and emergency admissions [14] and may not receive adequate care when the demand for nursing care exceeds supply. The rationing of fundamental nursing care and the associated development of potentially preventable AEs, such as those outlined in the F2M framework [8], is underpinned by the concept of missed nursing care which refers to aspects of nursing care that are regularly missed due to factors such as inadequate staffing [15].

Administrative healthcare data have been recognised as an efficient tool for monitoring rates of AEs and informing the development and evaluation of targeted prevention strategies [16]. Hospital discharge data in Ireland are an important indicator of hospital activity [17] and potentially an indicator of quality. The Hospital In-Patient Enquiry (HIPE) Scheme is a national information system for recording information on discharges and deaths from acute hospitals in Ireland [18]. The details of a patient’s care are documented in their clinical medical chart and on discharge, a discharge summary is created. Retrospective administrative data are generated as information is abstracted from chart documentation, such as discharge summaries, and assigned standardised codes by clinical coders using formal classification systems [19].

The classification system used in HIPE is the International Statistical Classification of Diseases and Health Related Problems, Tenth Revision, Australian Modification (ICD-10-AM) [20]. The statistics generated by this system inform epidemiological, quality assurance and market research. The Department of Health and the Health Service Executive in Ireland also use HIPE data to inform the planning, provision and monitoring of healthcare services in acute hospitals [17]. The Hospital Acquired Diagnosis (HADx) is a quality indicator used by HIPE since 2011 to identify and flag diagnoses acquired during the patient’s stay in hospital [20].

Whilst concerns over the accuracy of administrative datasets are evident [21–25], the potential of administrative data to provide accurate rates of AEs has been demonstrated [16, 26–35]. Other researchers recognise the potential of administrative data and acknowledge the need for further research to ensure that such datasets are accurate and robust [36–39]. A common method of measuring quality and safety in healthcare is through retrospective chart review [40]. Chart review has frequently been used to explore the accuracy of administrative data and identify whether it accurately reflects the occurrence of AEs in hospitalised patients [41, 42].

Previous literature has identified a research gap in using administrative data to inform patient safety improvements [16]. The aim of the present study was to validate HIPE data on the occurrence of pneumonia, urinary tract infections, pressure ulcers and delirium in older patients discharged from an acute hospital in Ireland through retrospective chart review.

## Methods

### Study design and setting

This research study is situated within the wider Cost2Care project which aims to demonstrate the potential for safer hospital care and reduced healthcare costs associated with older patients in acute hospitals in Ireland through routine measurement of missed nursing care, and sustainable and accurate reporting of nursing-sensitive adverse events using routinely collected hospital discharge data.

This was a retrospective cohort study using retrospective medical chart review and administrative healthcare data. A single university, tertiary hospital in Ireland with over 800 beds was selected for participation. In the year 2022, this hospital had over 100,000 patient presentations to the emergency department and over 25,000 inpatient discharges.

### Sample and study size

Admissions of inpatients aged 65 years and over who were discharged from 31 inpatient adult wards in the participating hospital between the 1 st of January 2022 and the 31 st of December 2022 were eligible for inclusion. Only charts of patients who were admitted for 72 h and longer were included to capture patients who experienced AEs, which are most likely to occur 48–72 h after admission [43, 44]. According to the Healthcare Pricing Office who oversee the national HIPE dataset, 6,575 admissions met these criteria in 2022 in the participating hospital. A simple simulation run in R using a range of AE rates identified that a sample size of 400 admissions abstracted gave an acceptably precise estimate. In order to support subgroup analysis, larger numbers were needed, therefore the sample was stratified into two categories of care homogeneity: medical and surgical admissions, consistent with DRG coding. The relative size of the groups, and the expected differences between them in terms of prevalence was not known. Therefore, a sample size of approximately 500 per category, giving 1,000 admissions in total would give reasonable power to detect moderately large differences between groups and allow for the exploration of the range of likely outcomes (Supplementary Fig. 1).

### Subject selection and data source

A random sample of admissions fulfilling the eligibility criteria was generated by the HIPE Department in the participating hospital. This sampling process resulted in a list of 1,000 admissions identifiable by Medical Record Number, episode number, admission date and discharge date. The participating hospital’s Medical Records Department made the charts available to the researchers during the chart review, in accordance with the ethical approval procedures described below.

### Co-production of chart review protocol and data collection instrument

A study-specific chart review protocol and data collection instrument were developed in collaboration with a public and patient involvement representative and representatives from the participating hospital to guide the chart review and data extraction process. These study materials were also developed in collaboration with project collaborator and retrospective chart review expert (MU), who provided a template for the primary and secondary review stages, and co-researcher (FB) from the Healthcare Pricing Office who provided expertise in relation to data protection considerations and the variables for inclusion in the primary review. A co-productive approach was then used to work with healthcare professionals from the participating hospital to further develop these study materials to tailor them to the context of the hospital. The Director of Nursing, Assistant Directors of Nursing (ADoN), an advanced nurse practitioner, a consultant geriatrician, a clinical nurse manager, a tissue viability nurse, a clinical nurse specialist, a consultant microbiologist and the hospital’s HIPE department manager were involved in the co-production of the chart review materials. An ADoN was appointed as coordinator of the research project in the participating hospital and facilitated this co-production process. Direct contact was made with the hospital representatives involved in the co-production process via meetings, arranged by this ADoN, where individual expertise was provided in relation to each of the AEs being investigated.

### Delirium

As the term “delirium” is not always used to document delirium in patients’ notes in the charts [45], various other terms such as “confusion” were deemed acceptable for identifying delirium through chart review, consistent with the literature [46]. This was agreed on consultation with relevant clinicians during the co-production process. Therefore, in this study, episodes of delirium were confirmed through the presence of the term “delirium” in addition to unconfirmed cases of delirium that were captured through the presence of alternative acceptable terms.

Due to the complex and interconnected relationship between delirium and dementia and the increased vulnerability/susceptibility of patients with dementia to developing delirium [47], patients with dementia were included. Patients who presented on admission with dementia who also experienced an episode of delirium, as per the chart review, had their pre-existing dementia documented during the secondary review.

### Pressure ulcer

A stage one pressure ulcer can be defined as intact skin with non-blanchable redness [48]. Based on this definition and communication with the healthcare professionals involved in the co-production process, documentation within the chart that denoted non-blanching redness was accepted as a stage one pressure ulcer. Stage 2, 3 and 4 pressure ulcers were recorded in the chart review when their staging was clearly documented in the clinical chart.

### Pneumonia and UTI

The World Health Organisation healthcare-associated infections case definitions were used to confirm the presence of pneumonia and UTIs throughout the chart review [49]. These definitions were provided to the research team by representatives from the participating hospital during the co-production process. These case definitions rely on radiology reports, symptoms and microbiology reports. A pneumonia or UTI was captured during the chart review if the patient met the criteria within the case definitions and was prescribed antibiotics to treat their condition.

### Inclusion and exclusion criteria of events in the chart review

The inclusion and exclusion criteria for each of the selected AEs are outlined in Table 1. Further information in relation to the inclusion and exclusion criteria for ICD-10 codes for identifying adverse events in the HIPE data can be found in supplementary Table 1.

**Table 1:** Inclusion and exclusion criteria for identification of adverse events in chart review

Adverse event	Inclusion criteria	Exclusion criteria
Pneumonia	Occurred 48 h after admission	Present on admission Occurred within 48 h of admission
Urinary Tract Infection	Occurred 48 h after admission	Present on admission Occurred within 48 h of admission
Pressure Ulcer	Occurred at any time after admission Deterioration of existing pressure ulcer	Present on admission with no worsening
Delirium	Occurred at any time after admission Deterioration of existing delirium	Present on admission with no worsening

### Review team pilot test and data collection process for chart review

The review team consisted of two reviewers: a clinical research nurse with previous chart review experience, and a PhD student. Both reviewers received training from the chart review expert (MU).

### Pilot

A pilot of the chart review was conducted in November 2023 to ensure that all relevant data were being collated and to confirm that the reviewers were able to locate the information using the online patient information systems and in the paper-based charts. A sample of 72 admissions were included in the pilot and the results of the pilot were communicated to the healthcare professionals involved in the co-production process. Following the pilot, the researchers met with a retrospective chart review expert (MU) for further educational training and to discuss the pilot results. The pilot results were retained in the main study as no major changes were made.

### Overall process

The retrospective chart review commenced in October 2023 and was completed in April 2024. The review of 1,000 admissions using paper-based charts took place on site at the participating hospital. Record notes from all clinicians within the clinical chart were reviewed. Access to the online patient information system was granted for reviewing laboratory results, imaging records, prescriptions and referral data. Additionally, access to the intensive care unit or the high dependency unit specific online systems was granted for patient admissions that had used these services.

A two-stage review process was used to conduct the retrospective chart review. The co-produced and agreed chart review protocol was used to guide the chart review process and included definitions, inclusion and exclusion criteria and step-by-step instructions on the data extraction process and the selected variables.

### Review stage 1

All 1,000 admissions included in the random sample were subject to Review Stage 1. There was no cut-off for maximum length of stay therefore, charts were reviewed for patients admitted for at least 72 h up until discharge. All notes pertaining to the admissions selected were screened by the reviewers. Admission and discharge times, number of bed and ward moves, and frailty scores were collected. If a potential AE was identified, the admission was forwarded to Review Stage 2.

### Review stage 2

Further information in relation to the identification and treatment of the AE, such as severity, antibiotic use, interventions and referrals were collected during review stage 2.

All abstracted data was entered into the study-specific data collection instrument using a spreadsheet in the Excel software. The data collection instrument consisted of a series of variables relating to the patients’ encounter with the hospital. The data was entered into Excel using either open-text or, for variables with standardised data, a drop-down menu with preset responses such as “yes” and “no”.

## Reliability and validity 

### Interrater reliability

Interrater reliability was determined by double review of 10% of the charts to assess the agreement between the chart reviewers’ decisions in relation to whether a chart was to be considered for secondary review, whether an AE was identified and whether the reviewer independently identified the same specific AE. The double review was overseen by a senior member of the research team (MK) who was external to the review team. Any discrepancies between the two reviewers and the senior member of the research team were discussed, and consensus was achieved. The consensus data was included in the data analysis for the double reviewed charts.

The review process was monitored by a senior member of the research team (MK) who ensured that the review was conducted in adherence to the guidelines outlined in the chart review protocol and was available for queries from the reviewers. Consistency between the reviewers was supported throughout the chart review as the reviewers shared a designated room on site at the participating hospital and were able to promptly discuss any queries. Any escalated queries were resolved via discussions between the reviewers and the senior member of the research team (MK). The chart review expert (MU) was also available for questions from the reviewers throughout the review process. 

## Analysis

The percentage of admissions that contained at least one AE was calculated in addition to the numbers of each of the AEs identified in the chart review. An AE was included in the HIPE data sample if a HADx flag was assigned to one of the predetermined ICD-10 codes in an admission. All included and excluded ICD-10 codes can be found in supplementary Table 1.

The chart review was conducted blindly as the reviewers did not have access to the corresponding HIPE data until after the chart review had been completed. On completion of the review, the HIPE data pertaining to each of the 1,000 admissions that were reviewed was received from the HIPE department at the participating hospital. The chart review data and corresponding HIPE data with procedure codes, diagnosis codes and HADx flags for each of the 1,000 admissions were merged in Microsoft Excel to allow for a comparison of the two data sources. Each AE identified in the chart review was investigated in the HIPE data to determine whether the event was assigned an ICD-10 code and a HADx flag. Although certain patients may experience multiple episodes of the same type of AEs, HADx data does not measure incidence, therefore the presence of the AEs is only coded for once in the HADx data. As a result, the comparisons between the HADx data and the chart review data were carried out at admission level. Furthermore, the number of AEs recorded in the HADx data but not identified through the chart review was also explored. The percentage of events that were identified in the chart review and were assigned HADx flags was also calculated.

Descriptive statistics such as frequencies were used. The sensitivities, specificities, positive predictive value and negative predictive values of the HADx data were calculated using the chart review as gold standard. Sensitivity represents the proportion of admissions with an AE identified during the chart review that were correctly recorded in the HIPE data using a HADx flag. Specificity represents the proportion of admissions without an AE according to the chart review that were correctly classified in the HIPE data without a HADx flag. The positive predictive value (PPV) represents the proportion of admissions that were captured in the HIPE data and confirmed by the chart review data. The negative predictive value (NPV) represents the proportion of admissions predicted by the HIPE data to not have an AE that were confirmed by the chart data to have not had an AE. Confidence intervals of 95% were set for sensitivity, specificity, PPVs and NPVs. Cohen’s Kappa and percentage agreement were calculated to establish inter-rater reliability between the reviewers. All statistical calculations were performed using IBM SPSS Statistics version 29.

## Results

### Participants

The study population consisted of 6,575 admissions of patients that met the inclusion criteria of being aged 65 and over with a length of stay of 72 h or longer and discharged in 2022. A sample of 1,000 admissions pertaining to 959 patients were included in the chart review. The demographics for the study cohort can be seen in Table 2.

**Table 2:** Patient demographics

Demographic variables	Total study cohort *n* = 1,000	Surgical admissions *n* = 505	Medical admissions *n* = 495
Gender			
Male, n (%)	534 (53.4%)	278 (55%)	256 (51.7%)
Female, n (%)	466 (46.6%)	227 (45%)	239 (48.3%)
Age, mean (SD)	77.2 (7.6)	76.3 (7.3)	78.1 (7.8)
Length of stay, days, median (min-max)	9 (3-200)	10 (3-200)	9 (3-172)
Type of admission			
Emergency, n (%)	769 (76.9%)	307 (60.8%)	462 (93.3%)
Elective, n (%)	231 (23.1%)	198 (39.2%)	33 (6.7%)

### Identified AEs and rates of AEs in Chart Review

In total, 373 AEs were found in 231 (23.1%) of the 1,000 reviewed admissions. There were 133 pressure ulcers, 101 cases of delirium, 84 cases of pneumonia and 55 UTIs identified by the chart review in 71, 100, 79 and 52 admissions, respectively. The 133 pressure ulcers occurred in 71 admissions with 62 secondary pressure ulcers (e.g. heel as well as sacrum) experienced in 27 of these admissions. Similarly, the 101 cases of delirium occurred in 100 admissions with one admission containing two episodes of delirium 16 days apart. The 84 cases of pneumonia occurred in 79 admissions as five admissions contained two episodes of pneumonia. The 55 UTIs occurred in 52 admissions with three admissions containing two UTIs.

**Fig. 1:**
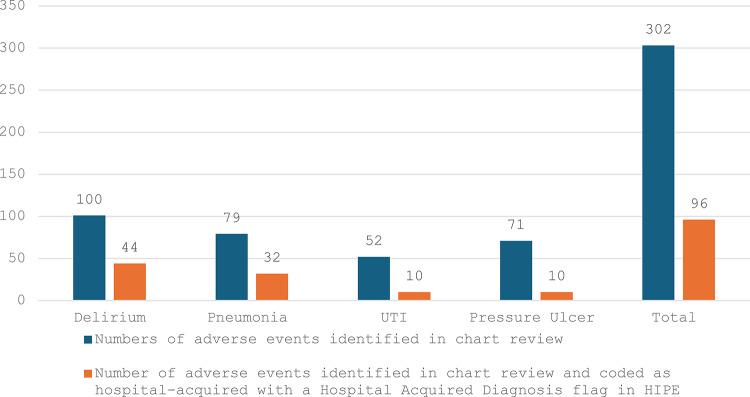
Admission level comparison between chart review data and HADx data

**Fig. 2:**
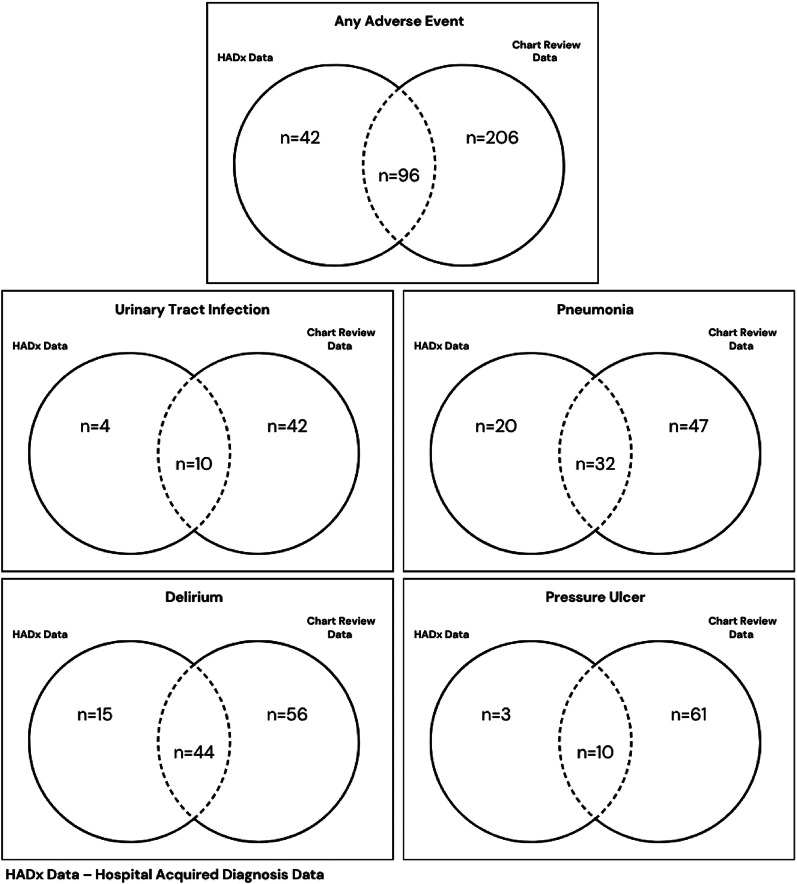
Adverse events as captured at admission level within chart review data and HIPE data

Since HADx data codes the presence of the respective AE once, the comparisons between the HADx data and the chart review data were carried out at admission level. Of the 302 AEs found in chart review on admission level, a HADx flag was found for 96 (31.8%) of the AEs. The AEs that had the highest representation of HADx flags, was delirium at 44%. The AE that the lowest representation within the HADx data was pressure ulcers, with 10 (14%) cases (Fig. 1). There were 42 AEs (20 pneumonias, 3 pressure ulcers, 4 UTIs and 15 delirium) in 38 admissions recorded in the HIPE data that were not identified through the chart review. The overlap between the chart review data and the HIPE data for AEs captured at admission level can be seen in the Venn diagrams in Fig. 2.

### Sensitivity,specificity,positive predictive value and negative predictive value

Overall, the sensitivity of the HADx data was low and the specificity was high. The PPVs of the HADx data varied whereas the NPVs were generally high (Table 3). The HADx data performed relatively well in relation to correctly identifying admissions without AEs however the results demonstrate that HADx did not accurately identify the AEs as captured through the chart review based on the lower sensitivity and positive predictive values.

**Table 3:** Sensitivity, specificity, PPV and NPV for HIPE data compared to chart review data at admission level

Type of adverse event	Sensitivity (95% CI)	Specificity (95% CI)	PPV (95% CI)	NPV (95% CI)	
Any adverse event	38.1 (32.0-44.5)	97.9 (9.67–98.8)	84.6 (76.9–90.7)		84.0 (81.5–86.3)
UTI	19.2 (10.1–30.3)	99.6 (99.0-99.9)	71.4 (45.5–90.1)		95.7 (94.4–96.9)
Pneumonia	40.5 (30.1–51.5)	97.8 (96.8–98.6)	61.5 (48.0–74.0)		95.0 (93.5–96.3)
Pressure Ulcer	14.1 (7.3–23.4)	99.7 (99.2–99.9)	76.9 (50.5–93.7)		93.8 (92.2–95.2)
Delirium	43.0 (33.6–52.8)	98.3 (97.4–99.0)	74.1 (62.0-84.2)		93.9 (92.3–95.4)

CI, confidence interval; NPV, negative predictive value; PPV, positive predictive value

### Inter-rater reliability

A total of 100 charts (10%) were double reviewed blindly by a third researcher. The inter-rater reliability of the reviewers’ judgements in relation to whether at least one AE was present at review stage 1, therefore indicating that a secondary review of the admission was to be carried out was κ = 0.740 and the percentage agreement was 90%. The inter-rater reliability of the reviewers’ judgements in relation to whether a specific AE was identified was κ = 0.793 with a 97% percentage agreement.

## Discussion

In this validation study, a total of 373 AEs within 231 admissions were identified at event level. Given that HADx data only codes for the presence of a condition once per admission, a total of 302 AEs with 100 episodes of delirium, 79 cases of pneumonia, 71 pressure ulcers and 52 UTIs were identified at admission level through the chart review. In relation to the 302 AEs found through the chart review, the HADx data matched to only 31.8% of these with 44 episodes of delirium, 32 cases of pneumonia, 10 UTIs, and 10 pressure ulcers assigned diagnostic codes and HADx flags. Overall, the sensitivity of the HADx data for identifying at least one AE of any of the studied AEs was low, but the specificity was high. The PPVs of the HADx data varied whereas the NPVs were generally high.

The AEs investigated in this study are nursing-sensitive patient outcomes which appear to be generally underreported in administrative data. Accurate reporting of these outcomes is essential for benchmarking and addressing rates of these specific AEs, but also for increasing the visibility of nursing within administrative data. Nursing care and the nurse workforce are vital to improving patient outcomes. Therefore, increased visibility of nursing-sensitive patient outcomes within administrative datasets is key to ensuring accurate information to guide and inform decision-making in the context of workforce planning which may improve patient outcomes [50].

The rates of AEs identified through this chart review are in line with international studies that estimate AE occurrence in 7-68.5% of admissions [2, 3]. The age profile of the patients in this study and their increased vulnerability to experiencing hospital-acquired conditions given the clinical complexity of their care is attributable to the rates of AEs identified in this study. Many previous studies have reported that older patients are affected by AEs to a higher extent than younger patients [51–53]. Older patients are particularly vulnerable to the four AEs explored in this study, as demonstrated in previous work [16, 53, 54].

The results of this study demonstrate a high level of variation between the rates of the four AEs captured through the chart review and the rates of these AEs as recorded in the HIPE data. Similar to the findings from previous chart review validation studies [21, 25, 55, 56], these findings suggest that the ICD-10 coding of these AEs within the HIPE data are a poor measure of hospital-acquired pneumonia, UTIs, pressure ulcers and delirium. Coding education is critical to maximising recognition and capture of common conditions. It is possible that recent changes in terminology have affected capture, for example, the change in nomenclature from pressure ulcer to pressure injury in some countries, though not globally. Coders benefit from interprofessional collaborative workshops, and also effective digital systems for information retrieval and reduced duplication [57].

The high variation in accuracy of discharge coding [58] has led researchers to explore the use of information technology to improve clinical coding accuracy and quality. Computer-assisted clinical coding has been reported to be valuable for improving on the accuracy, quality and efficiency of manual coding [59]. Although there are various challenges associated with introducing automated tools into clinical coding environments [60], their potential to enhance efficiency, quality and safety and reduce costs within healthcare settings is promising [59]. Artificial intelligence (AI) algorithms such as natural language processing (NLP) are successful methods of identifying AEs that can potentially be used to reduce the subjectivity of manual chart reviewing however, such methods were not feasible in this study given that the charts reviewed were paper-based. Although promising, AI tools are not without limitations and algorithms such as NLP should be validated using various sample sizes and settings [61].

Overall, the HIPE data had low sensitivity and high specificity in relation to the identification of the AEs investigated in this study. Similar results have been identified by Maass et al. [62] who found low sensitivity and high specificity when comparing ICD coding to chart review data. Caution is recommended when relying on hospital acquired complication flags in hospital data repositories, and in the quest for absolute activity-based funding approaches [63].

A notable finding was the identification of AEs in the HADx data that were not identified through the chart review. The variation between the inclusion criteria used for the respective AE by the chart reviewers and the inclusion criteria used by the clinical coders may have contributed partly to this divergence. Coding errors, which can contribute to administrative data inaccuracies [64], are also a potential source of variation in this study. Also, it is possible that human error, which is a well-acknowledged limitation of chart reviews [65], resulted in AEs being omitted.

Clinical coders must adhere to guidelines when abstracting information from patients’ clinical records. Due to the complex and multifaceted nature of coding clinical data, challenges such as unclear documentation, variation in terminology and legibility, incomplete charts, variability in coder interpretation and a high workloads with quota expectations can impact administrative data quality [19, 66–68]. Imprecise documentation may contribute to under-reporting of conditions in administrative data [19] due to unspecified diagnoses or an inability to code specific conditions. According to HIPE coding standards, coders cannot assign HADx flags to conditions where it is unclear in the clinical documentation if they arose during or prior to admission [69]. This may be cause for the low visibility of the four AEs in the HIPE data. Incomplete documentation may be driven by the time pressure that physicians are under to submit health information. Additionally, coders also experience time pressure when generating administrative data in a fast-paced environment with quota expectations. This limits their ability to devote excess time to re-checking charts with organisational issues [19]. To address this, clear and thorough documentation by clinicians of conditions that occurred during a patient’s admission is required.

The ability of HIPE data to capture hospital-acquired diagnoses using HADx flags demonstrates the potential of HIPE to accurately measure these four AEs. Although the rates at which HADx flags were assigned to the AEs identified through the chart review was low, improved documentation may make information more accessible to coders and therefore enhance the coding and visibility of these AEs within the HIPE data. As demonstrated by Chapman et al. [70] who found a moderate level of agreement between the clinical gold standard and coding processes for in-hospital stroke, this current study also highlights the possibility for HADx flags to accurately record hospital-acquired conditions with improved documentation and coding.

As highlighted by Hughes et al. [71], education of junior physicians, who are responsible for completing clinical documentation, on the importance of clear and accurate documentation is key to improving the visibility of hospital-acquired diagnoses and ensuring accuracy of hospital activity in HIPE data. Administrative burden on healthcare services in relation to clinical coding can also be alleviated through the streamlining of clinical documentation workflows [72]. Efficient clinical documentation workflows may provide a solution to the organisational issues that have been previously identified by coders [19]. Furthermore, collaborative workshops where clinicians and coders can share their perspectives have been shown to improve coders’ knowledge for classifying health conditions and improve clinicians’ understanding of the information required when documenting health conditions in clinical notes [57]. Improved accuracy will result in high quality data that can be used to inform research, improve patient safety and healthcare quality and ensure appropriate funding [17].

Although these findings indicate that hospital-acquired conditions may be underestimated in HIPE data, further research to improve the visibility of these AEs within HIPE data will allow for improved healthcare management, performance and planning. Improved visibility of nursing-sensitive patient outcomes may inform future research and decision-making in relation to nursing [50]. Furthermore, inaccurate data has a negative impact on hospital reimbursement [71] therefore, improved accuracy will further facilitate and enable accurate activity-based funding. Timon et al. [73] previously reported a funding deficit of €40,293 due to the underreporting of AEs in 23 patients. The economic impact of high quality and accurate reporting of AEs has been demonstrated by Lee et al. [74] who identified a total of €61,956 in missed funding for AEs following joint arthroplasty after only 15 AEs were identified in the HIPE data in comparison to 114 AEs which were identified through a prospectively implemented AE form. In addition to the considerable human cost of the four AEs explored in this study, the financial cost of these AEs is particularly burdensome for the health service [12, 13]. This highlights the importance of accurate administrative data for hospital reimbursement and further demonstrates the need to improve the visibility of AEs in administrative data. The economic cost of these adverse events will be further explored in future publications related to the wider Cost2Care project.

## Strengths and limitations

A strength of this study was the ability of the chart reviewers to discuss chart related queries by conducting the review side-by-side in a room at the participating hospital. Furthermore, although Cohen’s Kappa is commonly used to test interrater reliability, its limitations include that it may overly lower the estimate of agreement. Therefore, based on best practices [75], both the percentage agreement and kappa statistic have been used to measure the interrater reliability of the chart review. All interrater reliability kappa values were classified as substantial and percentage agreement scores were ≥ 90%, demonstrating the high quality of the review. The large sample size and random selection of charts for review reduced bias and enhanced the generalisability of the results [40].

The co-production of the data collection instrument and the study-specific chart review protocol with clear objectives and data extraction guidelines is another strength of this research which reduced variability and ensured accuracy, reliability and consistency [40, 76]. Additionally, ‘Co’ approaches are promising methods for improving healthcare [77] that allow academics and healthcare professionals to combine their skills in the research process [78] and provide a framework for bridging the gap between research and practice [79].

The reliability and validity of chart reviews can depend on the subjectivity of the review variables [76], such as the determination of pressure ulcers based on the amount of redness in an affected area [75]. Reduced agreement and validity are a limitation of subjective review variables however, the use of a chart review protocol and the ability of the reviewers to discuss any queries mitigated the risk of reduced validity due to the subjective nature of chart reviewing.

Furthermore, it is not known whether the 42 AEs identified only in the HIPE data were true or false positives as it was not feasible to gain access to the charts pertaining to these events once the chart review and HIPE data had been merged. While the potential explanations for these 42 AEs have been outlined, the uncertainty in relation to these events is a limitation of this study.

Lastly, the inclusion of only one tertiary university hospital is also a limitation of this study. Including different hospital models may have increased the generalisability of the findings however, conducting chart reviews at multiple hospital sites was not feasible due to labour and resource limitations.

## Conclusion

In conclusion, the variation between the chart review findings and the ICD codes demonstrates that HIPE data may not currently represent accurate rates of the four studied AEs. Improvements and education in relation to both coding and clinical documentation processes is necessary to allow the potential of these data as safety indicators to be fulfilled. Increased visibility of nursing within administrative data will contribute to improved patient outcomes as accurate measurement of nursing-sensitive patient outcomes may inform future research and nursing-related decision-making. Improved data accuracy will facilitate benchmarking of AE rates across hospitals and countries and provide opportunities to improve patient safety and healthcare quality and reduce the human and economic cost of AEs.

## Electronic supplementary material

Below is the link to the electronic supplementary material.

Supplementary Material 1.

Supplementary Material 2.

## Data Availability

The data used to inform this research is not publicly available due to data protection in ethics processes. As it was not possible to obtain the explicit consent of the individual to process their personal data, a consent declaration was received from the Health Research Consent Declaration Committee in Ireland to review the patient charts. This data cannot be shared outside of the research team.

## Declarations

### Abbreviations

ADoN Assistant director of nursing.

AE Adverse event.

AI Artificial intelligence.

CI Confidence interval.

F2M Failure to maintain.

HADx Hospital acquired diagnosis.

HIPE Hospital inpatient enquiry scheme.

ICD-10-AM International classification of diseases, 10th revision, Australian modification.

NLP Natural language processing.

NPV Negative predictive value.

PPV Positive predictive value.

UTI Urinary tract tnfection.
