# Supplementary material for: Validating adverse events in administrative healthcare data in Ireland: a retrospective chart review study
Source: BMC Health Serv Res. 2025 Aug 20;25:1113. doi: 10.1186/s12913-025-13201-x (PMC12369203; doi:10.1186/s12913-025-13201-x)
Supplement: Supplementary file 1 — Supplementary Material 1. [file 12913_2025_13201_MOESM1_ESM.docx]

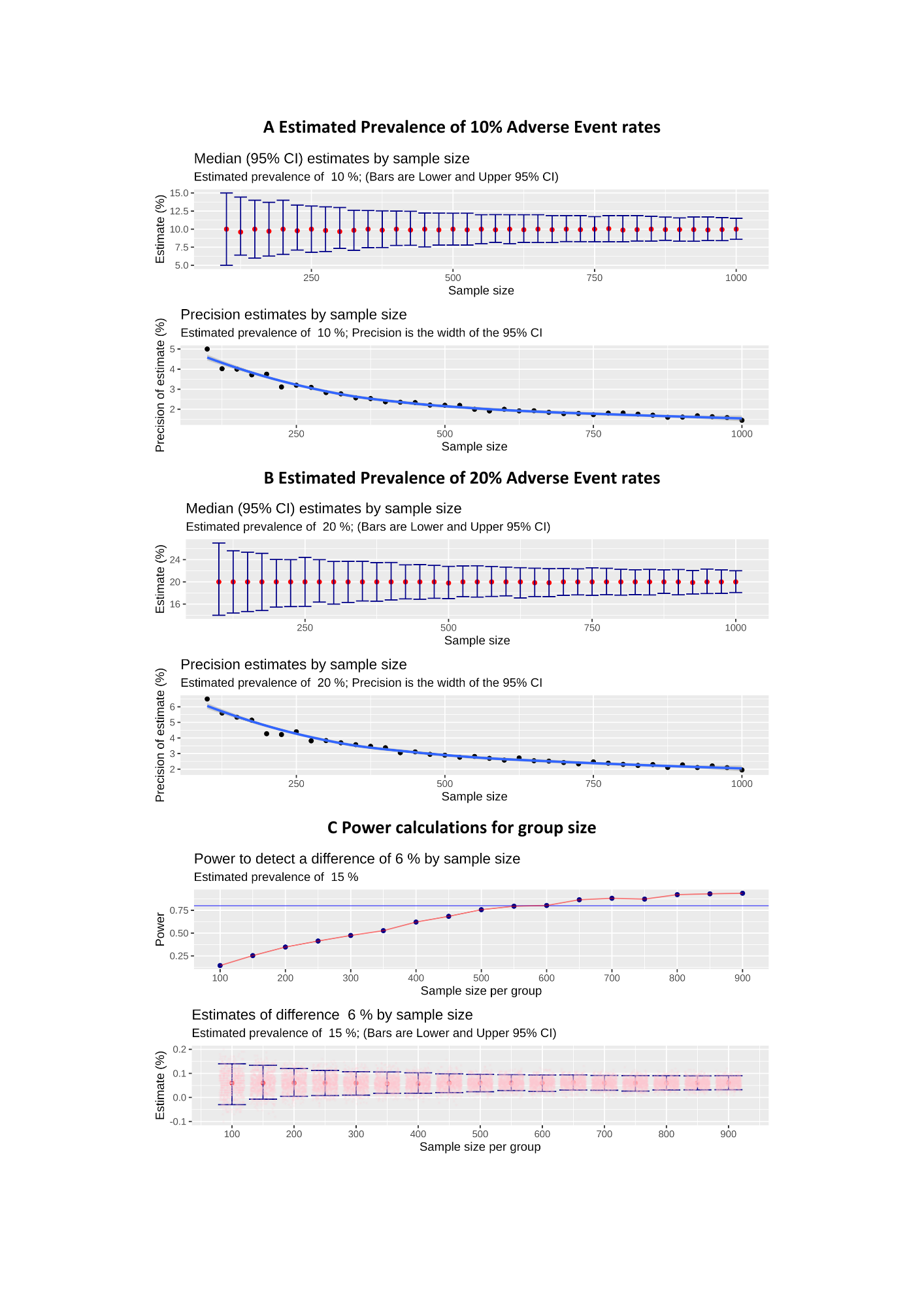


Supplementary figure 1: Sample size calculations. (A) Estimated prevalence of 10% adverse event rates, (B) Estimated prevalence of 20% adverse event rates, (C) power calculations for group size.
