# Supplementary material for: Validating adverse events in administrative healthcare data in Ireland: a retrospective chart review study
Source: BMC Health Serv Res. 2025 Aug 20;25:1113. doi: 10.1186/s12913-025-13201-x (PMC12369203; doi:10.1186/s12913-025-13201-x)
Supplement: Supplementary file 2 — Supplementary Material 2. [file 12913_2025_13201_MOESM2_ESM.docx]

Supplementary table 1: Inclusion and exclusion criteria for ICD-10 codes for identifying adverse events in the HIPE data

| **Inclusion Criteria** |  |  |
| --- | --- | --- |
| **Adverse event** | **Inclusion** | **Description** |
|  | ICD-10 Code (Secondary) |  |
| Urinary tract infection | N39.0 | Urinary tract infection, site not specified |
|  | T83.5 | Infection and inflammatory reaction due to prosthetic device, implant and graft in urinary system |
| Delirium | F05.0 | Delirium not superimposed on dementia, so described* |
|  | F05.1 | Delirium superimposed on dementia* |
|  | F05.8 | Other delirium* |
|  | F05.9 | Delirium, unspecified |
|  | R40.1 | Stupor |
|  | R40.2 | Coma, unspecified |
|  | *Recommended update (not in original Needleman approach) | |
| Pressure ulcer | L89^ | Pressure ulcer |
|  | ^Includes all subsets of L89, e. g., L89.0, L89.1, L89.2 etc. | |
| Pneumonia | J14 | Pneumonia due to Haemophilus influenzae |
|  | J15 | Bacterial pneumonia, not elsewhere classified |
|  | J18 | Pneumonia, organism unspecified |
|  | J69.0 | Pneumonitis due to food and vomit |
|  | J95.8 | Other postprocedural respiratory disorders |
|  | J95.9 | Postprocedural respiratory disorder, unspecified |
| **Exclusion Criteria** |  |  |
| Urinary tract infection | **Exclusion** | **Description** |
|  | ICD Code (Principal) |  |
|  | A40 | Streptococcal sepsis |
|  | A42 | Actinomycosis |
|  | A49.9 | Bacterial infection, unspecified |
|  | N39.0 | Urinary tract infection, site not specified |
|  | T83.5 | Infection and inflammatory reaction due to prosthetic device, implant and graft in urinary system |
|  | **Exclusion** | **Description** |
|  | Diagnose Code (Any) |  |
|  | O08.8 | Other complications following abortion and ectopic and molar pregnancy |
|  | O23.4 | Unspecified infection of urinary tract in pregnancy |
|  | O23.9 | Other and unspecified genitourinary tract infection in pregnancy |
|  | O86.2 | Urinary tract infection following delivery |
|  | O86.3 | Other genitourinary tract infections following delivery |
|  | **Exclusion MDC** | **Description** |
|  | 11 | Diseases & Disorders of the Kidney & Urinary Tract |
|  | 12 | Diseases & Disorders of the Male Reproductive System |
|  | 13 | Diseases & Disorders of the Female Reproductive System |
|  | 14 | Pregnancy, Childbirth & the Puerperium |
|  | 15 | Newborns & Other Neonates |
| Delirium | **ICD-10 Exclusion - Principal Diagnosis** | **Description** |
|  | F43.2 | Adjustment disorders |
|  | F43.9 | Reaction to severe stress, unspecified |
|  | F44.88 | Other specified dissociative [conversion] disorders |
|  | F05.9 | Delirium, unspecified |
|  | R40.1 | Stupor |
|  | R40.2 | Coma, unspecified |
|  | Exclusion MDC | Description |
|  | 1 | Diseases & Disorders of the Nervous System |
|  | 19 | Mental Diseases & Disorders |
|  | 20 | Alcohol/Drug Use & Alcohol/Drug Induced Organic Mental Disorders |
| Pressure ulcer | **ICD-10 Exclusion - Principal Diagnosis** | **Description** |
|  | L89 | Pressure ulcer |
|  | **ICD-10 Exclusion - Any Diagnosis** | **Description** |
|  | G80 | Cerebral palsy |
|  | G81 | Hemiplegia |
|  | G82 | Paraplegia and tetraplegia |
|  | G83 | Other paralytic syndromes |
|  | Exclusion MDC | Description |
|  | 9 | Diseases & Disorders of the Skin, Subcutaneous Tissue & Breast |
| Pneumonia | **ICD-10 Exclusion - Principal Diagnosis** | **Description** |
|  | J10 | Influenza due to other identified influenza virus |
|  | J11 | Influenza, virus not identified |
|  | J12 | Viral pneumonia, not elsewhere classified |
|  | J13 | Pneumonia due to Streptococcus pneumoniae |
|  | J14 | Pneumonia due to Haemophilus influenzae |
|  | J15 | Bacterial pneumonia, not elsewhere classified |
|  | J17 | Pneumonia in bacterial diseases classified elsewhere |
|  | J18 | Pneumonia, organism unspecified |
|  | J69.0 | Pneumonitis due to food and vomit |
|  | J95.8 | Other postprocedural respiratory disorders |
|  | J95.9 | Postprocedural respiratory disorder, unspecified |
|  | **ICD-10 Exclusion - Any Diagnosis** | **Description** |
|  | B20 | Human immunodeficiency virus [HIV] disease resulting in infectious and parasitic diseases |
|  | B21 | Human immunodeficiency virus [HIV] disease resulting in malignant neoplasms |
|  | B22 | Human immunodeficiency virus [HIV] disease resulting in other specified diseases |
|  | B23 | Human immunodeficiency virus [HIV] disease resulting in other conditions |
|  | B24 | Unspecified human immunodeficiency virus [HIV] disease |
|  | D80 | Immunodeficiency with predominantly antibody defects |
|  | D81 | Combined immunodeficiencies |
|  | D82 | Immunodeficiency associated with other major defects |
|  | D83 | Common variable immunodeficiency |
|  | D84 | Other immunodeficiencies |
|  | D86 | Sarcoidosis |
|  | D89 | Other disorders involving the immune mechanism, not elsewhere classified |
|  | M35.9 | Systemic involvement of connective tissue, unspecified |
|  | **Exclusion MDC** | **Description** |
|  | 4 | Diseases & Disorders of the Respiratory System |
